# UV exposure causes energy trade-offs leading to increased chytrid fungus susceptibility in green tree frog larvae

**DOI:** 10.1093/conphys/coac038

**Published:** 2022-07-03

**Authors:** Rebecca L Cramp, Michel E B Ohmer, Craig E Franklin

**Affiliations:** School of Biological Sciences, The University of Queensland, Brisbane, Queensland 4072, Australia; Department of Biology, University of Mississippi, MS, 38677, USA; School of Biological Sciences, The University of Queensland, Brisbane, Queensland 4072, Australia

**Keywords:** trade-off, pathogen, metabolism, energy, disease

## Abstract

Levels of ultraviolet (UV) radiation have increased in many parts of the world due to the anthropogenic destruction of the ozone layer. UV radiation is a potent immunosuppressant and can increase the susceptibility of animal hosts to pathogens. UV radiation can directly alter immune function via immunosuppression and photoimmunotolerance; however, UV may also influence pathogen defences by affecting the distribution of energy resources among competing physiological processes. Both defence against UV damage and repair of incurred damage, as well as the maintenance of immune defences and responding to an immune challenge, are energetically expensive. These competing demands for finite energy resources could trade off against one another, resulting in sub-optimal performance in one or both processes. We examined the potential for a disease-related energy trade-off in green tree frog (*Litoria caerulea*) larvae. Larvae were reared under high- or low-UV conditions for 12 weeks during which time we measured growth rates, metabolic rate and susceptibility to the amphibian fungal pathogen, *Batrachochytrium dendrobatidis* (*Bd*). We found that larvae exposed to high levels of UV radiation had higher rates of energy expenditure than those exposed to low UV levels; however, UV exposure did not affect growth rates or developmental timings. Larvae exposed to high UV radiation also experienced greater *Bd* infection rates and carried a higher infection burden than those not exposed to elevated UV radiation. We propose that the increased energetic costs of responding to UV radiation were traded off against immune defences to protect larval growth rates. These findings have important implications for the aetiology of some *Bd*-associated amphibian declines, particularly in montane environments where *Bd* infections are most severe and where UV levels are highest.

## Teaser

Ultraviolet (UV) radiation is a potent immunosuppressant. In addition to direct effects on immune pathways, increased solar UV radiation exposure can influence disease susceptibility in green tree frog larvae by causing energy trade-offs among competing physiological processes.

## Introduction

Solar ultraviolet (UV) radiation, in particular UVB (280–320 nm), is absorbed by DNA resulting in linkages between adjacent pyrimidine nucleotide bases (Diffey, 1991). In animals, UV-induced DNA damage can trigger immunotolerance in the skin (Spellman *et al.,* 1984) and can initiate an immunosuppressive cytokine cascade that culminates in systemic immunosuppression (Kripke *et al.,* 1992). Through its immunosuppressive effects, elevated UV can increase the susceptibility of animals to pathogens (Norval *et al.,* 2007; Cramp *et al.,* 2014; Abhimanyu, 2017). Increased solar UV radiation exposure has been hypothesized to have contributed to the emergence of the novel fungal pathogen, *Batrachochytrium dendrobatidis* (*Bd*), which causes the disease chytridiomycosis, and is responsible for the loss or decline of 500 amphibian species globally (Carey, 1993; Blaustein *et al.,* 2012; Cramp and Franklin, 2018). Importantly, chytridiomycosis emerged contemporaneously with anthropogenic increases in solar UV, and a number of significant disease-driven population declines occurred at high elevation where UV levels are highest (Blumthaler *et al.,* 1997; Kriger and Hero, 2008; Sola *et al.,* 2008). Despite being proposed over 25 years ago (Carey, 1993), a mechanistic link between elevated UV levels, immune suppression and increased pathogen susceptibility in amphibians has not been established.

**Table 1:** Absolute irradiance levels and cumulative daily doses of UVA and UVB radiation in the high- and low-UV treatment groups (values are mean ± s.d.)

Treatment	Intensity (W m^−2^)		Cumulative Daily Dose (kJ m^−2^)	
	UVB (290–320 nm)	UVA (320–400 nm)	UVB	UVA
High UV	0.27 ± 0.05	0.52 ± 0.09	5.75 ± 1.08	11.01 ± 1.88
Low UV	0.01 ± 0.04	0.131 ± 0.02	0.27 ± 0.8	2.75 ± 0.4

Amphibian embryos and larvae are highly susceptible to UV radiation (Blaustein *et al.,* 1994; Alton and Franklin, 2017). Although UVB can kill embryos and larvae outright (Kiesecker and Blaustein, 1995), low-level exposure can cause a suite of sub-lethal effects that impairs physiological performance (van Uitregt *et al.,* 2007; Alton *et al.,* 2010; Bernal *et al.,* 2011). Amphibian larvae possess a number of DNA protection mechanisms such as skin melanization (Alho *et al.,* 2010) and DNA repair (Morison *et al.,* 2020); however, they likely come at an energy cost (Alton *et al.,* 2012). In environments where energy resources are constrained, energy trade-offs occur when resources are directed towards physiologically pressing processes (such as responding to UV exposure) and away from less pressing processes (such as immune defence). The immune system is an energetically expensive physiological system that can trade off against other physiological processes such as reproduction (Schwenke *et al.,* 2016), wound healing and stress (Neuman-Lee and French, 2014). Physiological trade-offs are generally plastic and respond as needed to environmental variability (Schwenke *et al.,* 2016). Consequently, elevated UVB exposure may influence disease susceptibility indirectly by affecting the distribution of energy resources away from immune defence.

In this study, we investigated whether chronic, sub-lethal UV exposure increases the susceptibility of green tree frog (*Litoria caerulea)* larvae to infection by the fungal parasite, *Bd*. We hypothesized that exposure to elevated UV would lead to a trade-off between immune function and UV defence/repair, resulting in a greater susceptibility to *Bd* than larvae not exposed to high UV.

## Materials and methods

### Study animals and treatments

All experiments were conducted with the approval of the University of Queensland’s Animal Welfare Unit (SBS/085/13/URG) and Queensland Department of Environment and Heritage Protection (WISP12218412 and WISP16526515). Six green tree frog (*L. caerulea*) egg masses were collected from roadsides on Bribie Island, Queensland, Australia, the morning after laying and allowed to hatch overnight at room temperature in 50% site water and 50% filtered Brisbane tap water. Larvae were placed into individual 200-ml plastic containers containing 150 ml of carbon-filtered Brisbane tap water (8 cm deep). Containers were placed into a water bath at 23 ± 1°C to minimize thermal variability associated with UV lighting regimes. After 4 weeks, larvae were transferred into larger, 500-ml containers filled with filtered water to 75% capacity (5 cm deep).

Immediately after hatching, larvae were exposed to UV light generated by four 40 W full spectrum (UVB, UVA and visible wavelengths) fluorescent tubes (Repti Glo 10.0, Exo Terra, Montreal, Canada) positioned 50 cm above the water surface for 6 h per day. The low-UV treatment was shielded with commercial window tint (Energy Control Window Film, HandiHomes, Victoria, Australia), which blocked 96% of UVB and 75% of UVA wavelengths. Both treatments were also exposed to a 12 L:12D light cycle in the room generated by standard fluorescent light tubes. Peak irradiances generated by these treatment regimes were equivalent to a UV index of ~1.4 (high-UV group) and 0.4 (low-UV group) (Table 1). These values are considerably lower than peak UV levels in summer in Brisbane, Australia (UV index 14–16), and take into account the attenuation of UV by organic matter in natural water bodies and vegetation. Larvae were fed every second day with frozen spinach and 50% water changes were conducted on alternate days. All larvae were initially exposed to UV treatments for 12 weeks. After this time, a subset of larvae was removed for a *Bd* challenge experiment (which involved no further UV exposure). The remaining larvae were reared to metamorphosis in their respective UV treatments to determine the effect of UV exposure on larval duration.

### Mortality and growth rates

Immediately prior to the commencement of UV treatments, all larvae (36 per treatment) were photographed and total length, body and tail lengths were measured from images using ImageJ software (Schneider *et al.,* 2012). Body size morphometrics were collected weekly for 6 weeks, and then fortnightly until week 11 of exposure (prior to larval *Bd* exposure). For remaining larvae upon metamorphosis, defined as the point at which the tail was completely resorbed, time to metamorphosis (days), body mass and snout–vent length (SVL) were recorded. Larvae were checked daily and treatment-associated mortality events were recorded.

### Oxygen consumption rate

Resting metabolic rate, measured as the rate of oxygen consumption, was measured after 8 weeks of UV exposure as an indicator of the energetic cost of responding to UV radiation. Immediately after the day’s UV exposure, larvae (20 per treatment) were placed into individual 20-ml syringes containing an oxygen-sensitive sensor spot (5 mm, PSt3; Presens, Regensburg, Germany) and 20 ml of air saturated water. Syringes were sealed with a three-way tap and floated in a water bath maintained at room temperature (24 ± 1°C). Temperature-compensated oxygen partial pressures were measured non-invasively through the syringes 50–60 min later. Oxygen consumption rate ($\dot{V}{O}_2$; μg O_2_ h^−1^) was calculated using the equation

$$ \begin{align*} \dot{V}{O}_2=(\text{m}_{\text{a}}-\text{m}_{\text{c}}\times
V,) \end{align*}$$

where $\dot{V}{O}_2$ is the rate of oxygen consumption (μg O_2_ h^−1^), m_a_ is the slope of O_2_ consumption by the larvae over the trial (μg h^−1^), m_c_ is the slope of O_2_ consumption in a control (blank) respirometer over the trial (μg h^−1^) and V is the volume of water in the respirometer (l). Body mass was considered as a covariate in the statistical analysis.

### *Bd* infection, exposure and quantification

*Bd* strain EPS4 (Ohmer *et al.,* 2015) was maintained at 4°C in 0.5% tryptone-soy broth until 7 days before exposure. The strain was passaged onto 1% agar, 0.5% tryptone, 0.5% tryptone-soy plates and maintained at 21°C. After 7 days, zoospores were harvested by flooding plates with filtered tap water. The zoospore suspension was then collected and quantified using a haemocytometer following Boyle *et al.* (2004). A subset of larvae were removed from UV treatments after 12 weeks (8 per treatment; Gosner developmental stage 30) and transferred to an incubator set to 21°C. An aliquot of *Bd* zoospore isolate equivalent to ~250 000 zoospores was added to the water of each tadpole. Larvae were maintained for 1 week without water changes to allow the infection to establish. Thereafter, 50% of the water in tadpole containers was replaced every other day. Larvae were euthanized in 0.1% buffered MS222 after 4 weeks and the keratinized mouthparts removed for subsequent *Bd* quantification following Hyatt *et al.* (2007). Mouthparts were extracted in 50 μl PrepMan Ultra (Applied Biosystems, Foster City, CA, USA) and analysed in duplicate with quantitative PCR (Boyle *et al.,* 2004; Hyatt *et al.,* 2007) (MJ Mini Cycler, Bio-Rad Laboratories, Inc.). Ambiguous samples (one positive well, one negative well) were reanalysed in triplicate. A modified 15-μl reaction volume was used (Ohmer *et al.,* 2015) and infection load is reported as zoospore equivalents (ZEs).

### Statistical analyses

All statistical analyses were conducted using the statistical software package R (R Core Team, 2013). Growth rates were analysed using linear mixed effects (LME) models (*lmerTest* package; Kuznetsova *et al.,* 2013) with UV level (high or low) and weeks post-UV exposure as fixed effects and animal ID as a random factor to account for the correlated error from repeated measurements on the same animal. Survival rates were analysed using Cox proportional hazards regression model (*coxph* function in the *survival* package; Therneau and Grambsch, 2000). Size at and time to metamorphosis data were analysed using parametric and non-parametric *t*-tests. Metabolic rates were analysed using ANCOVA with UV level (high or low) as a fixed factor and body mass as a covariate. The effect of UV exposure level (high or low) on *Bd* infection prevalence was assessed using a Chi squared test. Infection intensity was compared using Mann–Whitney tests. For all tests, the threshold for significance was set at *P* < 0.05. All data are presented as means ± standard error unless otherwise specified.

## Results

### Developmental rates

**Figure 1:**
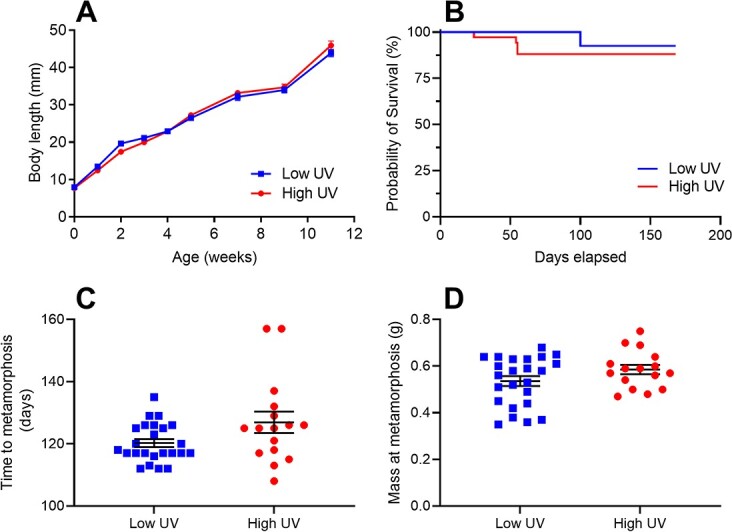
The effects of chronic, sub-lethal exposure to elevated UV radiation in larval green tree frogs (*L. caerulea*) on larval growth rates (as change in body length over time) (**A**), larval survival (**B**) and time to and mass at metamorphosis (**C** and **D**, respectively). Elevated UV exposure did not affect growth rates, developmental timing or body size metrics. Raw data are presented with mean ± SE error bars.

Larval development rates of *L. caerulea* were linear over time, and there was no significant effect of UV treatment (LME *F_(1,14)_* = 1.15, *P* = 0.31; Fig 1A). UV exposure treatments had no effect on *L. caerulea* larval survival (Cox PH: *z* = −0.961, *P* = 0.337, 95% *Conf* = 0.08, 2.38; Fig 1B). Neither body mass at metamorphosis nor time to metamorphosis were affected by UV rearing environment (mass: *t*-test, *t* = 1.6, *df* = 38, *P* = 0.11; time to metamorphosis: Mann–Whitney *U* = 133.5, *P* = 0.11; Fig 1C, D); however, snout to vent length was slightly greater in larvae exposed to high UV (*t*-test, *t* = 2.06, *df* = 38, *P* = 0.046).

### Oxygen consumption

**Figure 2:**
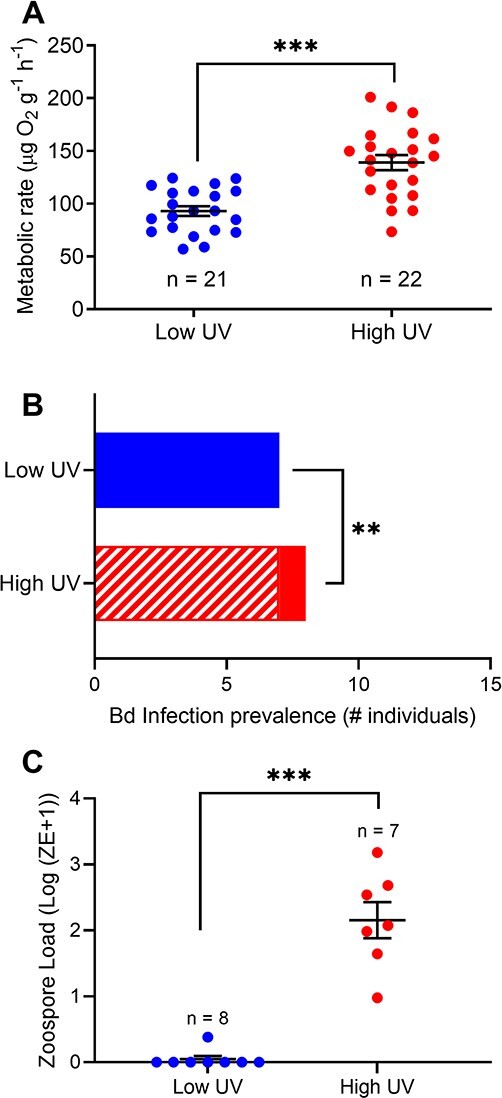
The effects of chronic sub-lethal exposure to elevated UV radiation in larval green tree frogs (*L. caerulea*) on whole animal metabolic rates (**A**) and the prevalence (**B**) and intensity (**C**) of infection by the fungal pathogen *B. dendrobatidis*. Exposure to high UV levels increased larval energetic costs and increased both the prevalence (infected, striped; uninfected, solid) and severity of pathogen infection. Raw data are presented with mean ± SE error bars. Asterisks indicate significant differences at ^*^P < 0.05, ^**^P < 0.01 and ***P < 0.001.

UV exposure regime had a significant effect on mass-corrected oxygen consumption rates (LM: *F_(2, 40)_* = 66.7, *P* < 0.001). Oxygen consumption rates in larvae from the high-UV group were almost 50% higher than in animals from the low-UV treatment (Fig 2A).

### *Bd* infection susceptibility and intensity

UV exposure had a significant effect on *Bd* infection rates with animals in the high-UV group experiencing higher rates of infection (100% infection in high-UV group vs 12.5% infection in the low-UV group; *χ^2^* = 8.24, *df* = 1, *z* = 2.87, *P* = 0.004; Fig 2B). Infection intensity was also significantly higher in the high-UV group (Mann–Whitney *U* = 0, *P* = 0.0003; Fig 2C). Infection intensities varied between 9 and 1500 ZEs for the high-UV treatment and between 0 and 2 ZEs for the low-UV group.

## Discussion

We show that chronic sub-lethal exposure to elevated UV levels increased larval energetic costs and *Bd* infection prevalence and intensity in laboratory-reared green tree frog larvae. UV is a potent inhibitor of both innate and adaptive immune function in mammals (Kripke, 1981) and fish (Jokinen *et al.,* 2000; Markkula *et al.,* 2009), but evidence to support a direct effect of UV on immune function traits in amphibians is lacking (Cramp and Franklin, 2018). UV exposure has previously been linked to higher embryonic mortality in the presence of a fungal pathogen (Kiesecker and Blaustein, 1995) and larval UV exposure reduced responses to an antigenic challenge in resulting juvenile frogs (Ceccato *et al.,* 2016). Our data show that exposure to relatively low levels of UV during the larval period increases *Bd* infection rates, suggesting that UV can influence disease susceptibility in larval and potentially adult amphibians by causing energy trade-offs.

Green tree frog larvae reared under high UV levels had substantially higher rates of oxygen consumption compared to larvae reared under low levels of UV, yet growth rates and size at and time to metamorphosis were not affected by the elevated UV exposure. This suggests that UV exposure results in higher metabolic costs in amphibian larvae, which may lead to an energy trade-off with the maintenance of another energy-demanding process—immune defences. DNA repair mechanisms (including photolyase or excision repair-mediated processes) are essential to ensure that UV-associated mutations do not accumulate, but these processes are energetically expensive to mount and sustain (Malloy *et al.,* 1997). The higher energetic costs associated with repairing DNA damage or mounting responses to limit UV damage (i.e. through increased skin pigmentation) could redirect finite energy resources away from the maintenance of basal immunocompetence or could limit the extent of response to a immunological challenge, both of which are also significant energetic costs (Lochmiller and Deerenberg, 2000; French *et al.,* 2009). That elevated UV exposure did not affect growth rates in *L. caerulea* suggests that growth rates may have been protected at the cost of immune function. Delaying the time to metamorphosis would increase the total UV received over the larval period, and protecting developmental rates would allow larvae to escape the challenging larval environment sooner (Brannelly *et al.,* 2019).

Although chytridiomycosis primarily affects the post-metamorphic life stages of amphibians, larval *Bd* infections are highly significant for amphibian populations. In larvae, *Bd* infections are restricted to the keratinized mouthparts and are not usually directly lethal. However, *Bd* infections can be carried through metamorphosis and can be a significant cause of mortality for juvenile frogs (Garner *et al.,* 2009). Metamorphosis is an immunologically challenging life stage in amphibians during which larval innate and adaptive immune pathways are significantly downregulated to prevent autoimmunity against the developing adult tissues (Rollins-Smith, 1998). In addition, the keratinization of juvenile frog skin expands the infective surface area for *Bd* colonization and contributes to the marked increase in *Bd* carrying capacity. Given that immune responses are significantly dampened as a result of metamorphosis, juvenile frogs are highly susceptible to *Bd* infections acquired during the larval phase (Garner *et al.,* 2009; Searle *et al.,* 2013). Additionally, elevated UV exposure could increase the tolerance of less-susceptible species to *Bd* infections (e.g. phototolerance; Spellman *et al.,* 1984), which may result in those species serving as a reservoir for *Bd* zoospores in the environment. Reservoir host species have been linked to several population declines in more susceptible amphibian species (Reeder *et al.,* 2012; Miaud *et al.,* 2016).

This study also highlights the complex and highly interconnected nature of physiological systems, which underpin the potential for physiological trade-offs to shape organismal responses to environmental change. Trade-offs are a particular challenge in conservation biology because they can make it difficult to predict the effects of environmental change on an organism. Trade-offs themselves are also not especially predictable (Smith and French, 2017). Moreover, the consequences of physiological trade-offs may span life history stages, manifesting well after the stressor(s) is/are experienced (i.e. carry-over effects). While trade-offs have long been a topic of interest in evolutionary biology, understanding when, where and why physiological trade-offs arise is increasingly important for how we study, assess and model complex animal responses to environmental change.

The data presented here provide a mechanistic link between exposure to elevated UV and an increased risk of *Bd* infection in amphibians. Given the highly varied nature of immune system responses to environmental stressors (Cramp and Franklin, 2018), further work is required to understand the specific mechanisms by which UV exposure influences disease susceptibility in larval amphibians. These findings have important implications for the aetiology of some *Bd*-associated amphibian declines, particularly in montane environments where *Bd* infections are most severe and where UV levels are highest (Blumthaler *et al.,* 1997; Kriger and Hero, 2008; Sola *et al.,* 2008).

## Data availability

All data are available via the UQ eSpace repository and are freely available for reuse with appropriate attribution (https://doi.org/10.48610/8e4a7f3).
